# Distribution of metal contamination and grain size in the sediments of Nakdong River, Korea

**DOI:** 10.1007/s10661-020-08475-z

**Published:** 2020-07-09

**Authors:** Shin Kim, Deuk Seok Yang, Yong Seok Kim

**Affiliations:** Nakdong River Environment Research Center, National Institute of Environmental Research, 24-11, Gukgasandan-daero 52-gil, Guji-myeon, Dalseong-gun, Daegu, Republic of Korea

**Keywords:** Nakdong River, Sediment, Grain size, Metal contamination

## Abstract

To assess distribution of metal contamination and grain size in the sediments of Nakdong River (South Korea), surface sediments were collected from 21 sites and analyzed. Within the study area, sand was typically the dominant grain size. However, because of the reduced flow rate and flow velocity, sites adjacent to weirs were composed of relatively fine sediments. A comparison of sediment metal concentrations with sediment quality guidelines proposed by the USA, Canada, and South Korea revealed that sites adjacent to weirs had concentrations that exceeded the standard values. The enrichment factor, index of geo-accumulation, and pollution load index calculation results that the sites adjacent to weirs showed high contamination, with Cd accounting for the highest contamination levels. The metals in the study area varies due to the effect of fine sediments; therefore, high concentrations of metals accumulated adjacent to weirs where fine sediments were distributed in greater proportions. Furthermore, Cd exhibited the greatest contribution to metal contamination in the study area and the highest contamination levels were found at NS19 (adjacent to the Haman weir). Thus, the accumulation of fine sediment increased due to the influence of the weirs, thereby increasing the overall amount of metal contamination.

## Introduction

A river is classified into a mainstream and tributaries, both of which are directly related to human activities. In recent years, the natural purification capability of rivers has decreased and the river environment has gradually deteriorated due to increased residential and industrial sewage and wastewater linked to population growth, improved living standards, and industrial progress (Kim et al. 2015a, b). Furthermore, river environments are largely affected by artificial structures, such as weirs, as well as cities and industrial complexes built adjacent to rivers (Ahn et al. 2014).

Contaminants flowing into a river are discharged to the environment through various paths. Contaminants flowing into the water system typically accumulate in sediments transported and deposited by the flow of water, waves, tidal currents, and wind. Contaminants deposited in rivers or lakes then accumulate on the bottom and influence the river ecosystem. To obtain a complete understanding of the river environment, it is important to analyze the geochemical components (e.g., metals) that accumulate in the sediments as well as the water quality environment (Thornton 1983). Water quality analysis is key for understanding the current short-term environmental conditions; however, sediments contain higher metal concentrations and exhibit smaller temporal and spatial changes than waster because of their limited movement. Therefore, river sediments are a useful tool for evaluating continuous environmental effects (Ra et al. 2013). Specifically, metals in sediments always exist in the aquatic environment and have an important influence on benthic organisms. Moreover, they have harmful effects on the hydro-ecosystem when released into the water and can lead to physical/chemical changes (Alloway et al. 1988; Dekov et al. 1997). Thus, determining the distribution and behavior of chemical components in sediments, including metals, reveals the sediment environment of a river and can be used to provide efficient response measures, such as controls on various environmental factors (Kim et al. 2001).

Previous studies on river sediments in South Korea have involved various analysis methods and evaluations of organic matter and heavy metals inside the sediments, with a particular focus on core and surface sediments (Kim et al. 2010; Park et al. 2011; Kim et al. 2015a, b). In addition, researchers have evaluated contamination levels using the concentration of metals distributed in the sediments. These studies typically assess the contamination level by comparing with the absolute baseline values of sediment quality guidelines (SQGs) derived for each country. Another typical assessment method employs the concentration of metals among crustal materials or in an uncontaminated area as the background concentration for comparison (Sekabira et al. 2010; Kim and Um 2013; Md et al. 2015; Han et al. 2016).

Unlike conventional studies that are performed within a limited spatial area, this study aims to investigate the complete sediment environment of the Nakdong River by determining the grain size distribution and metal contamination in the mainstream and tributaries of the Nakdong River system. Moreover, to assess the contamination level of metals, a comparison is made not only with the SQGs of South Korea but also with those of USA and Canada. Based on the measured metal concentrations, we calculate the EF (enrichment factor), Igeo (index of geo-accumulation), and PLI (pollution load index). Then, by assessing metal contamination at each site, we determine the area with the highest contamination for further analysis. Furthermore, the principal component analysis (PCA) method is used to determine the relationships between each variable and the main factors influencing the contamination level of sediments in the study area. As a result, the objective of this study is to understand distribution of metal contamination and grain size in sediments and provide useful data for future management and contamination assessments of river sediments.

## Material and methods

### Study area and sediment sampling

The study area, i.e., the Nakdong River in South Korea, has the basin area of 23,384.21 km^2^, a mainstream river length of 400.7 km, and a river length of 510.36 km. It is located in the southeast of South Korea at a longitude and latitude of 127° 29′ 19″–129° 18′ 00″ and 34° 59′ 41″–37° 12′ 52″. It lies adjacent to the Han River basin to the north and the Geum River and Seomjin River basins to the west (Jung et al. 2016). The Taebaek Mountains form the east sea coast basin and watersheds in the east, and the southern sea area of the Nakdong River lies to the south. The administrative areas include three metropolitan cities (Busan, Daegu, and Ulsan Metropolitan Cities) and parts of five provinces (Gyeongsangnam-do, Gyeongsangbuk-do, Jeonlanam-do, Jeonlabuk-do, and Gangwon-do). Furthermore, during the Four River Refurbishment Project, which was conducted in order to control floods and secure water resources that were deemed insufficient for the continuously increasing water demands of recent years, river channels were dredged and a total of 16 multi-purpose weirs were constructed. Of these, eight were built in the Nakdong River (NIER 2017).

**Fig. 1:**
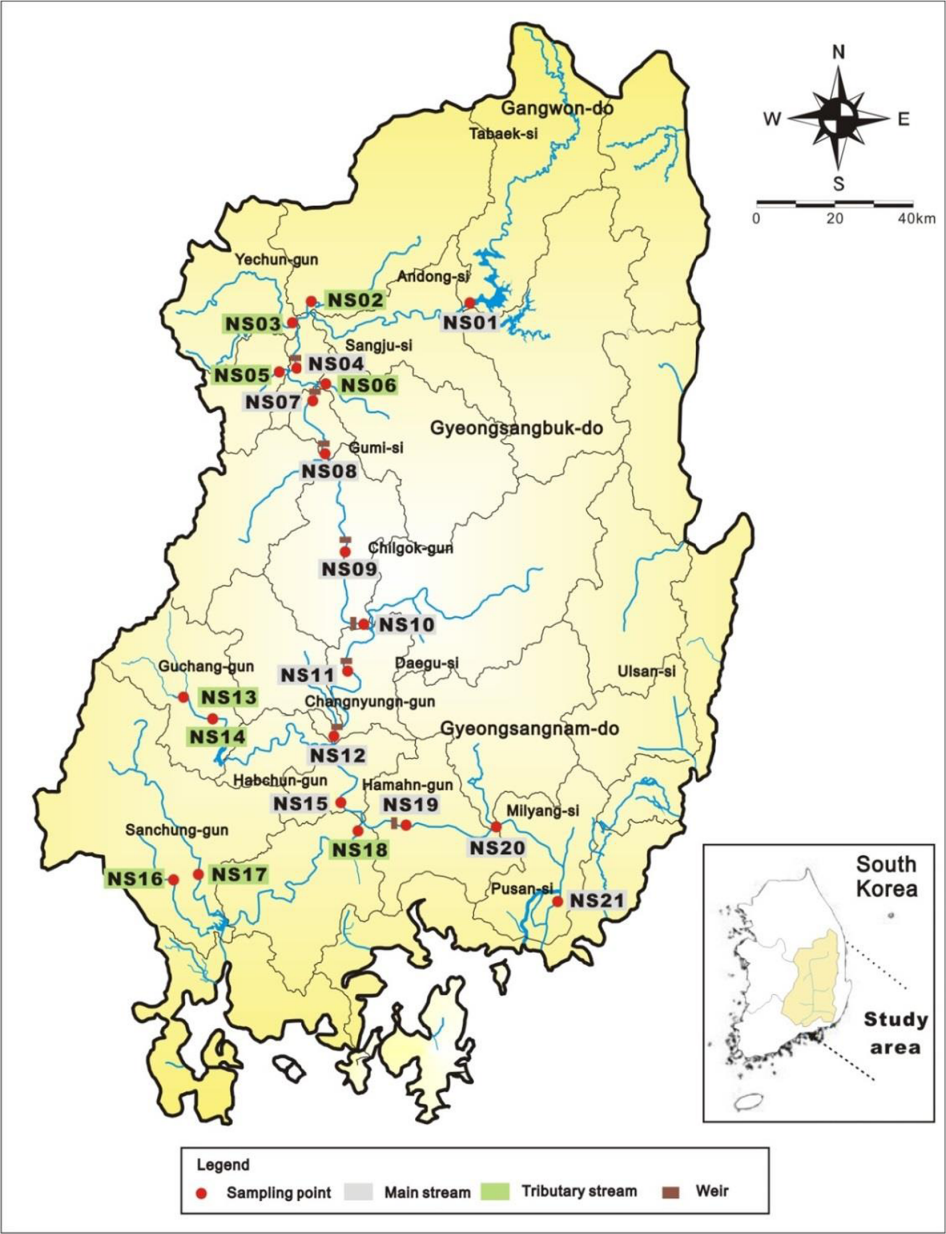
Location of sampling sites in the study area

This study selected a total of 21 sites in the mainstream and tributaries of the Nakdong River and collected surface sediments from September to November 2016. For the sediment samples, a Ponar grab was used, a type of gravity corer in which the bottom blade closes when the corer touches a basal surface, releasing the tension. Surface sediments were collected in this way from the upper 1–3 cm of sediments. Among the 21 sites, 11 are located in the mainstream and 10 in the tributaries. Eight of the mainstream sites (NS04, NS07, NS08, NS09, NS10, NS11, NS12, and NS19) are adjacent to weirs (Sangju Weir, Nakdan Weir, Gumi Weir, Chilgok Weir, Gangjeong-Goryeong Weir, Dalseong Weir, Hapcheon-Changnyeong Weir, and Changnyeong-Haman Weir, respectively) (Fig. 1).

### Analysis of surface sediments

The samples used for grain size analysis were first collected in plastic bottles and then transported to the laboratory for analysis. For the grain size analysis, a pre-treatment process was performed, which decomposed the organic matter with hydrogen peroxide (H_2_O_2_). Subsequently, the grain size was measured by a Microtrac S3500 grain size analyzer that uses the laser diffraction principle and calculates the grain size distribution by measuring the difference in diffraction patterns when the sediment grains pass through a laser beam. The measured results were classified into sand, silt, and clay according to the sediment composition. The sand was sub-divided into five sizes: very coarse sand, coarse sand, medium sand, fine sand, and very fine sand. The textural parameters for the sediments, such as mean grain size, sorting, and skewness, were obtained using the method of Folk and Ward (1957) after converting the weight percentage in each grain size class of sediments. To express the grade of grain size, because the logarithmic scale is more useful than the equimultiple scale, *Φ* (phi) was used, and the relation between *Φ* and grain diameter, *D*, was expressed as *Φ* = − log_2_ *D* (size in mm).

Samples for metal analysis (Al, Li, Zn, Cr, Pb, Ni, Cu, and Cd) in sediments were first mixed onsite at the time of collection using a non-metallic sample spoon and then sieved with a 150-μm sieve. Subsequently, samples were placed in glass bottles and stored in a portable cool box and were then transported to the laboratory for analysis. They were then dried in a natural state and crushed to obtain ground powder samples. The powder samples were pre-treated by adding HNO3, HClO4, and HF (in a 2:1:2 ratio), and the analysis was performed by an inductively coupled plasma–atomic emission spectrometry (ICP-AES). This analysis method was performed in accordance with the “test standard for sediments of river and lake” one of the “water quality pollution test standards” of the National Institute of Environmental Research of South Korea (Ministry of Environment 2012).

### Sediment quality guidelines

In this study, the metal analysis results were compared with the sediment quality guidelines of USA, Canada, and South Korea (Table 1). The US EPA sediment quality standard is a standard of the Regional Environmental Protection Agency for freshwater sediment contamination classification. According to the content of each metal element (Zn, Pb, Cu, Ni, and Cd), there are three standard classifications: non-polluted, moderately polluted, and heavily polluted (US EPA 1999). Canada’s Ontario sediment guidelines express the adverse effects of sediments on benthic organisms in terms of probability, with three contamination levels: NEL indicates no effect on the organisms living in the sediments, LEL indicates no effect on a large number of organisms living in the sediments, and SEL indicates an adverse effect on benthic organisms (CCME 1995). The National Institute Environmental Research sediment pollution evaluation of South Korea provides four levels of classification for the effects of sediment metal content on benthic organisms. Level I indicates almost no possibility of toxicity appearing in the benthic organisms. Level II indicates a possibility of toxicity, level III indicates a relatively high possibility of toxicity, and level IV indicates a very high possibility of toxicity (NIER 2015).

**Table 1:** US EPA sediment quality standard, Ontario sediment quality guidelines and NIER sediment pollution evaluation standard (unit: mg/kg)

	US EPA sediment quality standard			Ontario sediment quality guidelines		NIER sediment pollution evaluation standard			
	Non-polluted	Moderately polluted	Heavily polluted	LEL	SEL	I	II	III	IV
Zn	< 90	90–200	> 200	120	820	≤ 363	≤ 1170	≤ 13,000	> 13,000
Pb	< 40	40–60	> 60	31	250	≤ 59	≤ 154	≤ 459	> 459
Cu	< 25	25–50	> 50	16	110	≤ 48	≤ 228	≤ 1890	> 1890
Cr	-	-	-	-	-	≤ 112	≤ 224	≤ 991	≤ 991
Ni	< 20	20–50	> 50	16	75	≤ 40	≤ 87.5	≤ 330	> 330
Cd	-	-	> 8.0	0.6	10	≤ 0.4	≤ 1.87	≤ 6.09	> 6.09

### Calculation methods of EF, Igeo, and PLI

To evaluate metal contamination in sediments, comparisons are typically made with the metal contents of crustal material or natural concentrations in an uncontaminated area near the study area. These methods can estimate the concentrated amount of measured metal contents by using the difference or proportion of established standard elements and natural metal contents (Kim and Jang 2014). Accordingly, to eliminate the effect of grain size in the sediments, this study used Al, which is a major element in the sediments and exhibits relatively small content variations and a large distribution in the sediments. For the background concentrations, we used the values provided by the River Sediments Background Concentrations of the NIER of South Korea (NIER 2011), which are average concentrations of metals distributed in South Korean river sediments, considering the local characteristics of the study sites. The contamination level was then assessed by calculating the EF, Igeo, and PLI. The EF is calculated by Eq. (1):

1 $$ \mathrm{EF}=\left(\frac{M\left(\mathrm{metal}\right)}{M\left(\mathrm{reference}\right)}\right)\mathrm{sediment}\kern0.5em /\left(\frac{M\left(\mathrm{metal}\right)}{M\left(\mathrm{reference}\right)}\right)\mathrm{reference}\ \mathrm{value} $$

where (*M*(metal) / *M*(reference)) sediment is the concentration ratio of the metal to the reference element (Al) in the sediment sample, and where (*M*(metal) / M(reference)) reference value refers to the ratio of the metal to the reference element in the background concentration (Bruland et al. 1974). An EF value of 1.5 or less indicates an uncontaminated natural environment, whereas EF greater than 1.5 implies artificial contamination due to inflow through the air and river (Zhang and Liu 2002; Hyun et al. 2007). And Yongming et al. (2006) suggest deficiency to minimal metal enrichment when EF values are lower than 2.

The Igeo shows the relative concentration level of metals. Unlike the load coefficient, it can be used to evaluate the level of contamination by directly grading the contamination level of sediments. Igeo = 0–1 indicates unpolluted/moderately polluted, 1–2 indicates moderately polluted, 2–3 indicates moderately polluted/strongly polluted, and > 3 indicates strongly polluted. Igeo is calculated by Eq. (2), where *M*(sediment) is the corresponding metal concentration, *M*(background) is the background concentration of the corresponding metal, and a constant of 1.5 is multiplied for compensation (Muller 1979).

2 $$ \mathrm{Igeo}={\log}_2\frac{M\left(\mathrm{sediment}\right)}{M\left(\mathrm{background}\right)\times 1.5}\kern0.75em $$

The PLI can assess the overall contamination in an area, including all analyzed metals, unlike Igeo and EF. It is calculated by Eq. (3), calculated as a ratio of the metal concentration, *M*(sediment), and the background concentration of the natural environment state *M*(background). When PLI is higher than 1, it implies contamination. As the value increases, the contamination level increases (Tomlinson et al. 1980).

3 $$ \mathrm{PLI}=\sqrt[n]{\left(\frac{M\left(\mathrm{sediment}\right)}{M\left(\mathrm{reference}\right)}\right)1\times \left(\frac{M\left(\mathrm{sediment}\right)}{M\left(\mathrm{reference}\right)}\right)2\times \dots \left(\frac{M\left(\mathrm{sediment}\right)}{M\left(\mathrm{reference}\right)}\right)n\ } $$

### Principal component analysis

Principal component analysis is a statistical analysis method that groups multiple variables with similar common dimensions and reduces them to a relatively small number of factors (Grant 1990). This is the most common multivariate statistical method used in environmental studies to reduce data and extract a small number of latent factors for analyzing relations among the observed variables (Chen et al. 2007). To investigate the relationship between all factors analyzed in this study, grain size analysis (i.e., sediment composition, mean grain size, sorting, and skewness), analysis of the concentration and contamination (i.e., EF, Igeo, and PLI) of metals in the study area, and principal component analysis were conducted using SPSS 20.0 software.

## Result and discussion

### Distribution of grain size

**Fig. 2:**
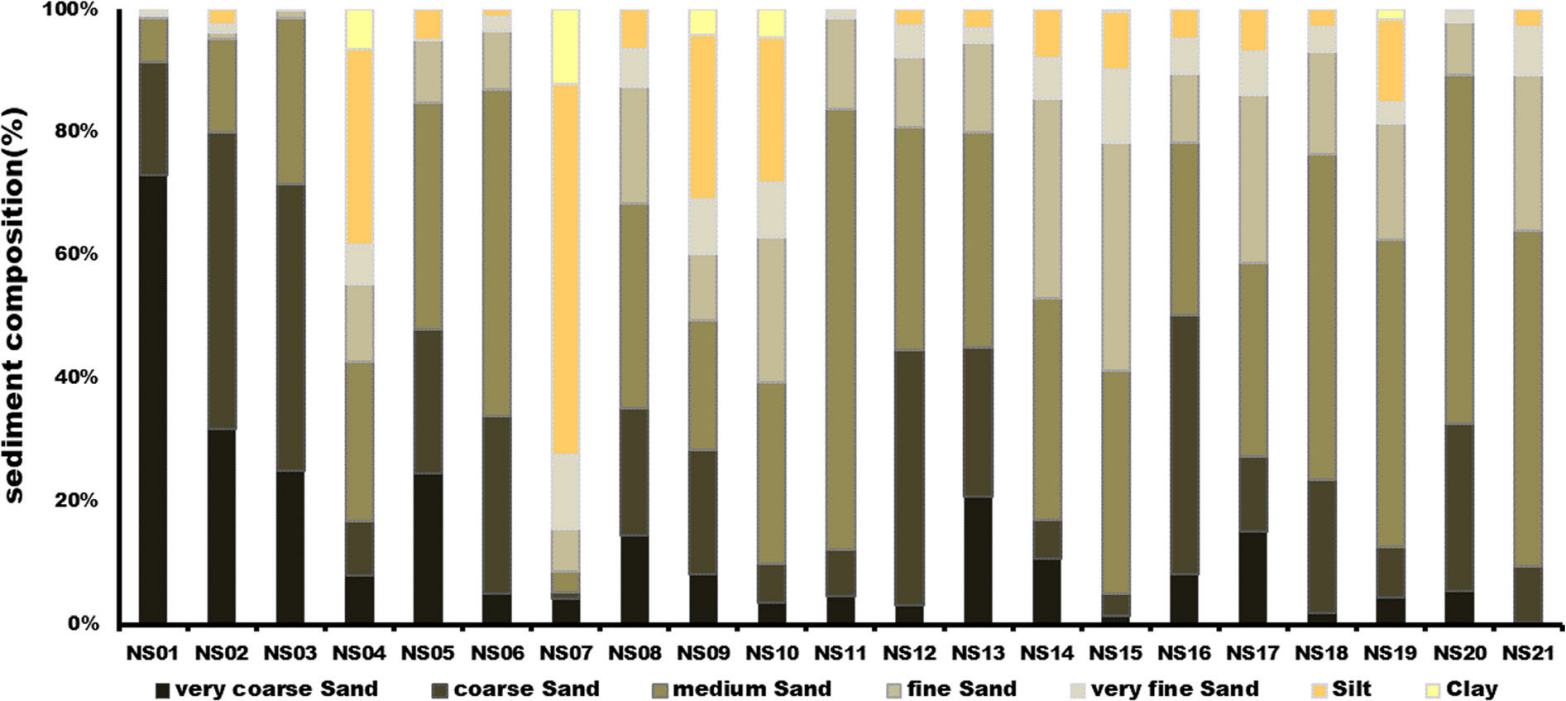
Sediment composition of surface sediments in the study area

According to the grain size analysis results of surface sediments in the study area, sand is the dominant grain size overall (average 88.02%). At the majority of sites, the sand content exceeds 90% with medium sand the most dominant class (average 34.96%). At sites NS04 (61.7%), NS09 (69.1%), and NS10 (71.9%), the areas adjacent to weirs constructed in the study area, slightly smaller sand contents are observed compared with other sites. Specifically, site NS07 is predominantly composed of fine-grained sediments, with 27.5% sand, 60.2% silt, and 12.1% clay (Fig. 2). Grain size is significantly influenced by the flow rate and flow velocity (Kim et al. 2017). Indeed, flow velocity changes and temporary stagnation of flowing streams are linked to the riverbed slope and river channel direction changes caused by the installation of artificial structures (Oh et al. 2003). This agrees with our results, whereby finer grain sizes are found at sites adjacent to weirs than at other sites because of the effect of decreased flow rate and flow velocity close to the weirs.

**Fig. 3:**
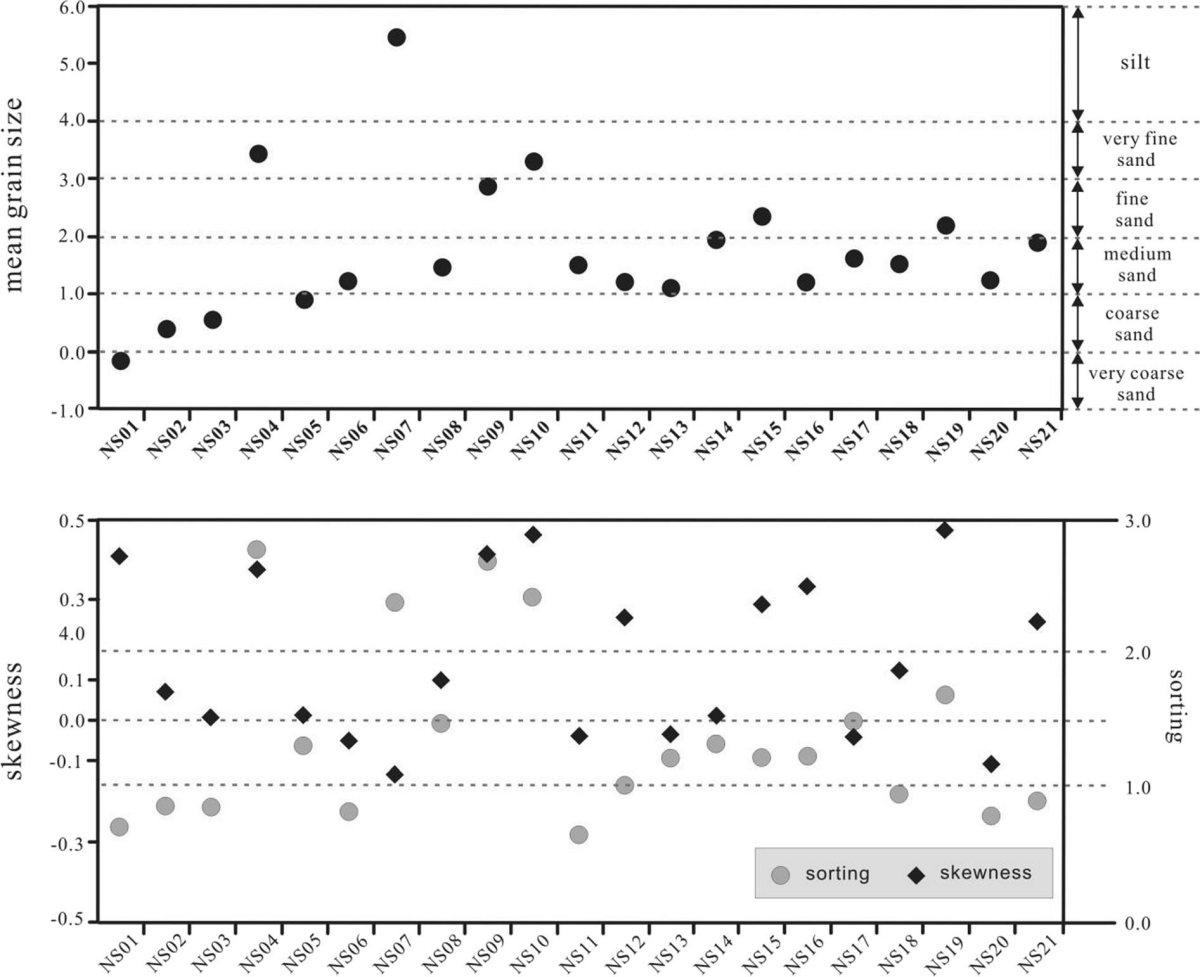
Mean grain size, sorting, and skewness of surface sediments in the study area

The mean grain size is 1.80 *Φ*, which corresponds to medium sand, and the sand grains are predominantly distributed in the ranges of 0–1 *Φ* (coarse sand) and 1–2 *Φ* (medium sand). However, at sites NS04 and NS07, where silt is dominant, the mean grain size is 3.43 *Φ* and 5.44 *Φ*, respectively, which are finest grain sizes in the study area. Sorting indicates the homogeneity level of grains through a standard deviation of the grains. A value of less than 0.35 indicates very well sorted grains, 0.35–0.5 *Φ* indicates well sorted, 0.5–1 *Φ* indicates moderately well sorted, 0.7–1.0 *Φ* indicates moderately sorted, 1–2 *Φ* indicates poorly sorted, 2–4 *Φ* indicates very poorly sorted, and above 4 *Φ* indicates extremely poorly sorted. The average value for the study area is 1.40 *Φ*, which corresponds to poorly sorted grains. In general, grains are moderated sorted and poorly sorted, whereas sites NS04, NS09, and NS10 correspond to very poorly sorted grains. Skewness shows the asymmetry of grain distributions and indicates the existence or non-existence of coarse and fine fractions. As the skewness approaches 0, coarse and fine sediments are distributed more symmetrically. The average value is 0.16 for the study area, and positive values are observed at all sites except for six that exhibit negative values (Fig. 3).

The textural parameters derived from the grain size analysis reflect the energy conditions in the sedimentation environment. In a low-energy environment, the sediments are fine, sorting is poor, and positive skewness is shown. Conversely, in a high-energy environment, coarse sediments with good sorting tend to show positive or negative skewness (Kim et al. 2018). Furthermore, a large positive value of skewness indicates dominant erosion due to physical effects such as flow velocity. On the other hand, when the skewness is small, the area can be classified as one where sedimentation is dominant because the physical effects are relatively weak (Folk 1980). In other words, the very high silt content adjacent to the weirs in the study area, as well as the poor sorting and low skewness, indicates a reduced flow rate and flow velocity, which can have large effects on the transport of sediments, thereby indicating a low-energy environment where sedimentation is active.

### Contamination and concentration of metal

The concentrations of the eight analyzed metals (Al, Li, Zn, Pb, Cu, Cr, Ni, and Cd) are shown in Table 2. The average concentration of Al is 6.9% and exceeds 8% at four sites. The average concentration of Li is 25.6 mg/kg, exceeding 40 mg/kg at three sites. The average concentration of Zn is 120.2 mg/kg and exceeds 200 mg/kg at NS09 and NS19. The average concentration of Cr is 48.5 mg/kg and is highest at site NS19 (83.6 mg/kg). The average concentrations of Pb and Cu are 24.6 mg/kg and 16.9 mg/kg, respectively. The highest concentrations of Pb (over 40 mg/kg) and Cu (43.3. mg/kg) are observed at site NS19, respectively. The average concentrations of Ni and Cd are 16.08 mg/kg and 0.38 mg/kg, respectively, and their maximum concentration is observed at site NS19.

**Table 2:** Metal concentrations of surface sediments in the study area

	Al (%)	Li (mg/kg)	Zn (mg/kg)	Cr (mg/kg)	Pb (mg/kg)	Cu (mg/kg)	Ni (mg/kg)	Cd (mg/kg)
NS01	7.98	12.0	160.0	11.6	14.8	11.4	8.0	0.72
NS02	7.29	22.2	82.2	12.4	23.0	10.5	10.2	0.12
NS03	4.62	21.9	64.3	36.3	20.8	9.2	12.6	0.17
NS04	7.70	29.2	118.5	44.3	25.4	37.9	17.5	0.36
NS05	4.79	11.6	67.0	29.5	18.2	9.5	10.0	0.19
NS06	4.74	20.7	49.0	52.1	18.0	8.5	11.6	0.32
NS07	6.76	42.3	145.3	55.8	30.4	25.3	27.0	0.39
NS08	7.34	36.0	137.1	50.5	27.2	19.7	20.9	0.39
NS09	6.39	42.1	217.1	51.8	31.9	29.4	28.3	0.42
NS10	8.54	30.9	120.4	50.5	26.6	18.2	19.0	0.46
NS11	4.17	17.2	81.6	62.4	23.8	13.7	11.2	0.53
NS12	5.23	19.3	75.6	49.2	22.5	10.9	12.1	0.52
NS13	8.14	21.2	87.6	23.6	22.5	8.5	4.8	0.26
NS14	7.13	28.6	151.4	29.2	23.8	13.9	8.2	0.22
NS15	5.71	24.7	120.4	62.6	22.3	13.3	15.7	0.36
NS16	8.30	17.8	86.0	72.5	17.6	10.2	17.5	0.34
NS17	8.06	25.6	127.7	79.9	24.4	20.7	26.3	0.34
NS18	7.17	19.6	62.1	61.6	18.7	9.8	13.9	0.29
NS19	8.25	43.9	213.1	83.6	35.5	43.3	32.4	0.51
NS20	6.97	22.4	95.9	56.9	25.6	8.5	13.5	0.45
NS21	7.61	26.7	126.7	28.0	28.0	12.2	15.3	0.40

Table 3 presents the comparison of the sediment metal concentration results of the study area (i.e., Nakdong River) with the results of Korea’s other three major rivers: Han River, Geum River, and Yeongsan River. As shown in the table, the metal concentrations in the sediments of the Nakdong River, i.e., all study sites for this investigation, showed similar results as the metal concentrations observed in the Geum River (Lee et al. 2014) and Yeongsan River (Shin et al. 2015). However, sediments of the Han River, which lies more closely adjacent to urban areas in comparison with the other rivers, showed relatively high metal concentrations, including Pb and Cu, demonstrating high pollution by these contaminants (Lai et al. 2013). Additionally, metal concentrations of river sediments found in other countries, such as China, India, and Australia, generally showed higher concentration distributions in comparison with this study area. In particular, the Ganga River of India can be seen to have significantly higher concentrations in comparison with the Nakdong River due to the effects of domestic, industrial, and agricultural untreated wastewater (Song et al. 2011; Duodu et al. 2016; Pandey et al. 2014). As such, it can be inferred that the sediments found in the Nakdong River showed lower metal concentrations in comparison with other river sediments due to the lesser amount of anthropogenic impacts due to domestic, industrial, and agricultural environmental factors.

**Table 3:** Comparison of different metal concentrations in surface sediments in this study and other studies

Location	Nation	Reference	Zn (mg/kg)	Cr (mg/kg)	Pb (mg/kg)	Cu (mg/kg)	Ni (mg/kg)	Cd (mg/kg)
Nakdong River	South Korea	This study	49.0–217.0	11.6–83.6	14.8–35.5	8.5–43.3	4.8–32.4	0.12–0.72
Han River	South Korea	Lai et al. (2013)	52.1–690.7	27.3–146.8	17.1–106.2	5.1–158.5	8.8–57.5	0.05–1.32
Geum River	South Korea	Lee et al. (2014)	57.5–124.9	37.6–78.6	8.3–19.4	11.8–22.4	9.9–20.9	0.05–0.18
Yongsan River	South Korea	Shin et al. (2015)	22–167	2–72	14–40	2–26	3–35	0.01–0.09
Brisbane River	Australia	Duodu et al. (2016)	142–257	82–332	25–126	20–110	20–34	0.6–0.9
Chanjiang River	China	Song et al. (2015)	50.6–221.0	64.5–126.7	19.0–173.2	10.8–87.9	24.2–52.6	0.35–16.45
Ganga River	India	Pandey et al. (2014)	137.3–201.2	126.8–196.1	148.8–211.4	12.7–84.0	14.6–82.5	9.5–79.0

Table 4 shows the comparison of metal concentrations in the study area with the US EPA sediment quality standards, Canada’s Ontario sediment guidelines, and South Korea’s NIER sediment pollution evaluations. According to the US EPA sediment quality standard, ten and two sites are moderately polluted with Zn and Pb, respectively. Moreover, sites NS09 and NS09 are heavily polluted for Zn and Pb, respectively. With respect to Cu and Ni, no sites are heavily polluted, but six and ten sites are moderately polluted, respectively. According to the Ontario sediment guidelines, one, five, and seven sites correspond to LEL for Zn, Pb, and Cu, respectively. Furthermore, for Cr, all sites excluding the four positions corresponding to NEL correspond to LEL. For Ni, 13 sites correspond to LEL. For Cd, all sites excluding NS01 correspond to NEL. According to the NIER sediment pollution evaluation, the majority of positions correspond to level I, which is an unpolluted level. However, at site NS09, Pb corresponds to level II, and at four sites (NS01, NS07, and NS08), Cd corresponds to level II, which is the highest contamination level among all the metals. In the comparative analysis with the SQGs of three countries, the majority of metal contents indicate high contamination levels at sites adjacent to weirs.

**Table 4:** Evaluation for sediment quality guidelines (SQGs) using US EPA, Ontario of Canada, and NIER of Korea

SQGs		Site name (metal)
US EPA	Non-polluted	Other sites (metals)
	Moderately polluted	NS01, 03, 10, 14, 15 (Zn), NS08 (Zn, Ni), NS07, 08, 09, 17, 19 (Zn, Cu, Ni), NS21 (Zn, Pb, Cu)
	Heavy polluted	N/D
Ontario of Canada	NEL	Other sites (metals)
	LEL	NS14, 15 (Zn), NS16 (Ni), NS01 (Zn, Cd), NS03 (Cu, Ni), NS07, 08, 10, 17 (Zn, Cu, Ni), NS09, 19, 21 (Zn, Pb, Cu, Ni)
	SEL	N/D
NIER of Korea	I level	Other sites (metals)
	II level	NS01, 09, 10, 19, 20 (Cd)
	III level	N/D
	IV level	N/D

**Fig. 4:**
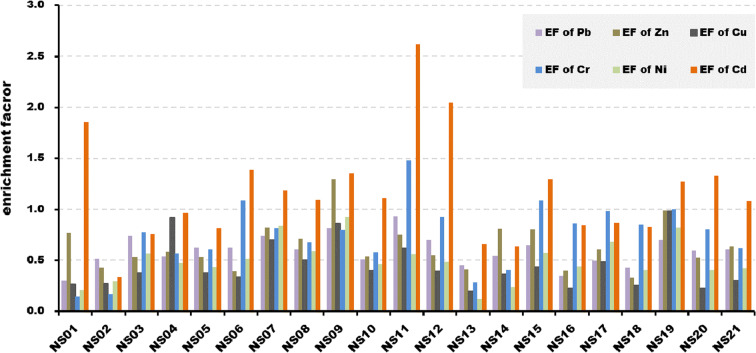
Enrichment factors of the metals of surface sediments in the study area

The EF calculation of surface sediments in the study area reveals Pb, Zn, Cu, Cr, and Ni values of less than 1.5 at all sites, indicating no anthropogenic contamination and minimal metal enrichment. However, Zn relatively higher contamination at site NS09 (1.30), adjacent to the Chilgok Weir. Moreover, Cr values are relatively higher at four sites, and Cd, the metal with the highest EF values, exhibits values over 1.5 at all except NS01, NS12, and NS13(adjacent to the weir), indicating that anthropogenic contamination is pervasive. These results imply that, in general, contamination is relatively high and large impacts in weir-constructed areas. Among all metals, contamination by Cd is the highest (Fig. 4).

**Fig. 5:**
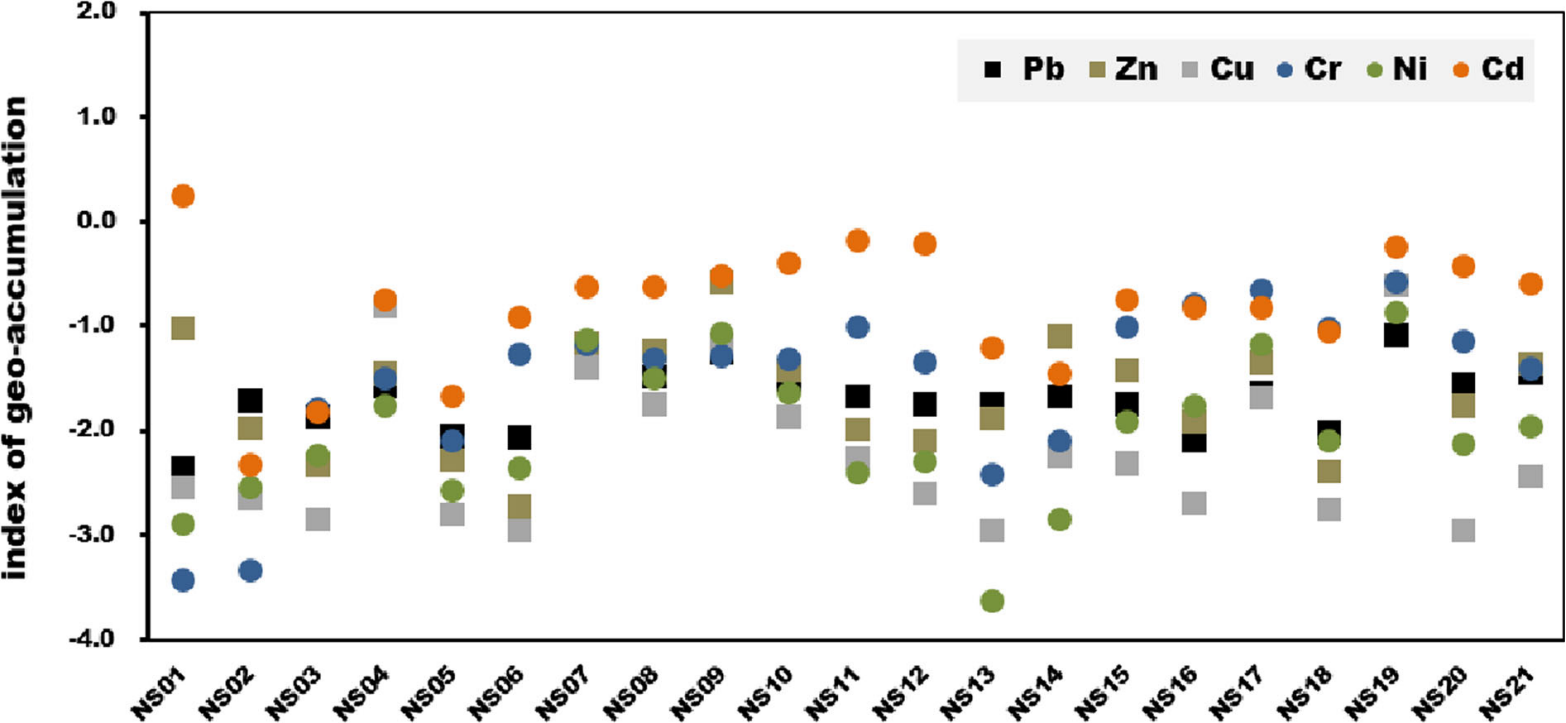
Index of geo-accumulations of metals of surface sediments in the study area

The Igeo value for Cd is 0.26 at site NS01, which corresponds to partially unpolluted/moderately polluted. Besides this, no other metals or sites exceed Igeo = 0, indicating that the contamination level is practically unpolluted. However, in the calculation results, Zn exhibits relatively high values at sites NS09 (− 0.57) and NS22 (− 0.34), and Cu and Cr exhibit relatively high values (− 0.60 and − 0.58, respectively) at site NS19. Because these results show similar trends to the EF results, we confirm that relatively high contamination appears at sites adjacent to weirs (Fig. 5).

According to the calculation results of EF and Igeo values, Cd was found to be relatively higher than other metals, indicating a state of higher pollution. Ganugapenta et al. (2018) previously suggested that the high contamination degree of Cd is generally attributed to agricultural runoff, industrial activities, and other anthropogenic inputs. Additionally, the official Group of Experts on the Scientific Aspects of Marine Pollution (GESAMP) ( 1985) proposed that the contamination degree of Cd may also increase due to influential factors, such as agricultural soils, mining waste, and municipal sewage effluents and sludges. Accordingly, it was assumed that Cd in sediments found in the NS01 (not adjacent to weirs) showed a relatively high contamination (EF > 1.5, and Igeo > 0) degree due to anthropogenic impacts. Therefore, additional studies should be conducted to explore detailed causes affecting the Cd concentration levels of sediments observed in the Nakdong River.

**Fig. 6:**
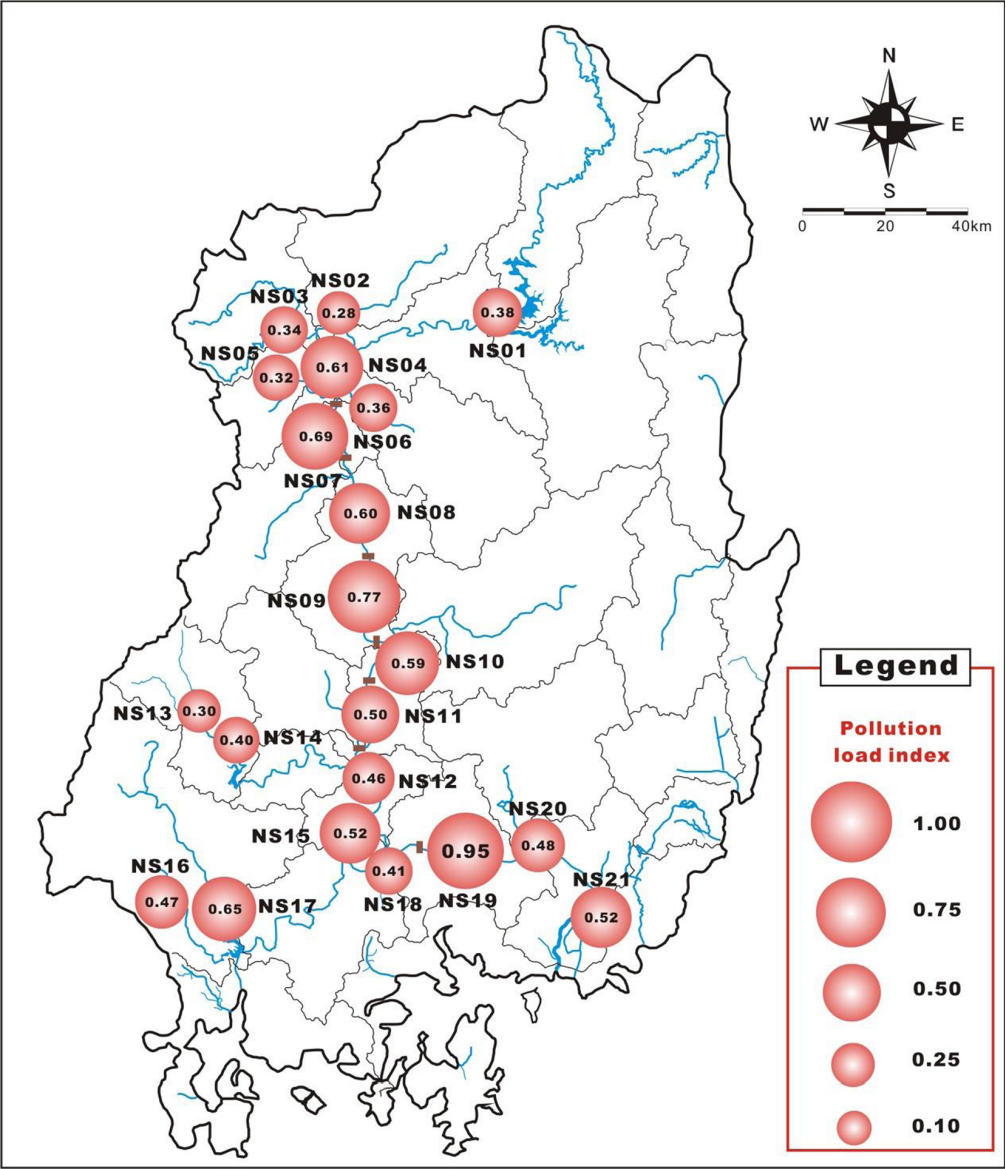
Pollution load index of surface sediments in the study area

Unlike the contamination assessments performed using EF and Igeo, the PLI value can be used to identify the total contamination level by using the concentrations of all analyzed metals. All PLI values for all sites in the study area are less than 1, indicating significantly low contamination. Furthermore, referring to the findings of one study, which suggested that the area with contaminated river sediments (PLI > 1) was influenced by metal pollutants from nearby mines, cities, and industrial activities (Ahn et al. 2019), the PLI value of the present study area was shown to be considerably low, indicating that for this area, the anthropogenic impacts on metals induced by nearby areas were relatively small. However, PLI values also indicate relatively high contamination at the sites NS04, NS07, NS08, NS09, and NS19, which are located in the weir sections. Among these sites, metal contamination is highest at site NS19 (PLI = 0.95), located at Haman Weir (Fig. 6).

### Principal component analysis

The principal component analysis was performed using the EF, Igeo, and PLI calculation results, which were employed in the grain size, metal concentration, and contamination assessment, as the factors analyzed in this study. As a result, two principal components were extracted. In the principal analysis results, the eigenvalue, which indicates how much each factor explains the information of existing variables, is 46.08% for factor 1 and 15.08% for factor 2.

**Fig. 7:**
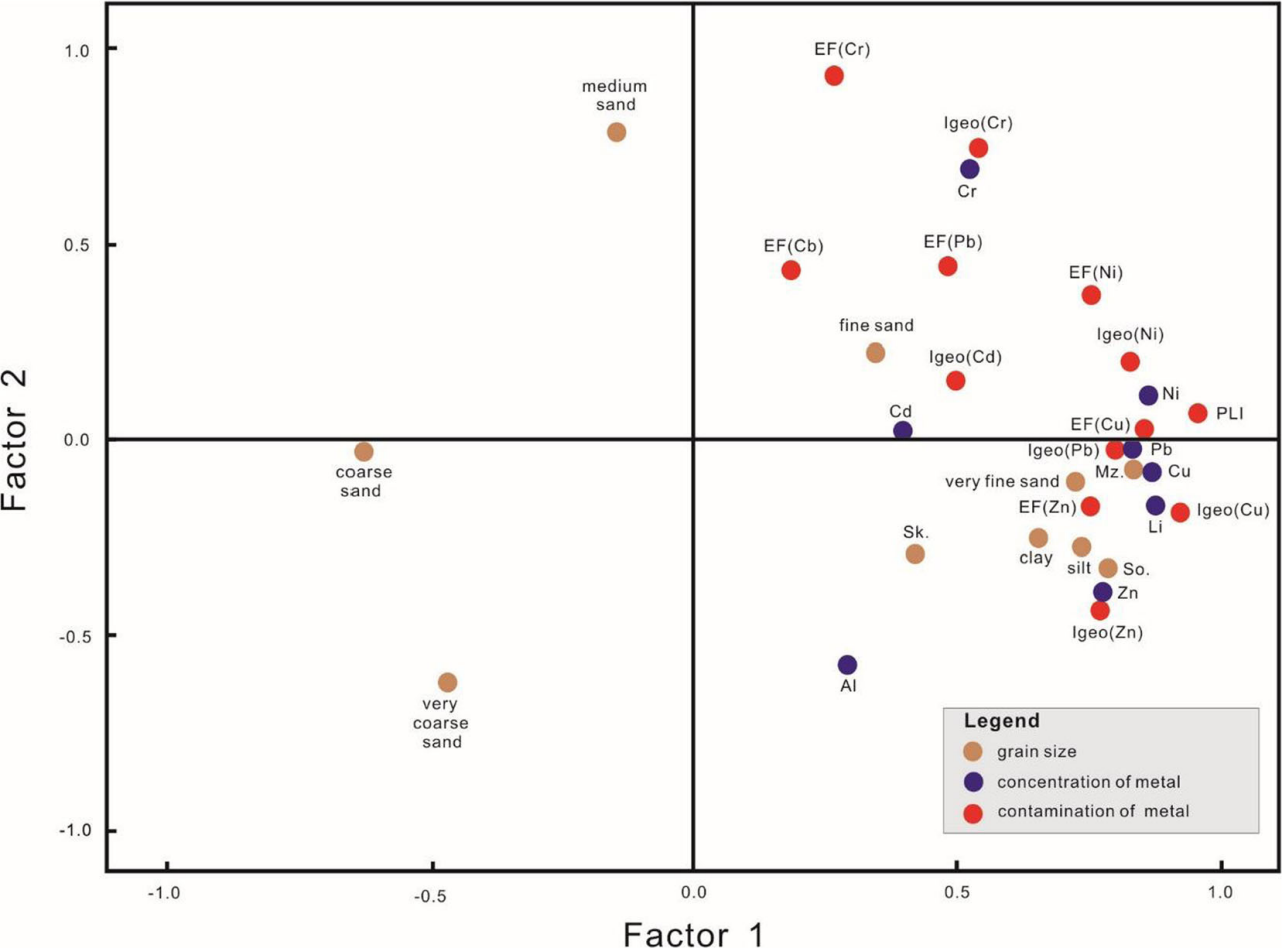
Plot of principal component analysis of surface sediments in the study area

The factors corresponding to factor 1, which have the largest effect on the sediment environment of the study area, are PLI, Zn, Pb, Cu, and Ni, EF, Igeo, fine sediments such as very fine sand, silt, and clay, and mean grain size (Table 5). In the component diagram that visualizes the principal components, Zn, Pb, Cu, and Ni, which are the metals that have no effect on metal contamination, are concentrated on the right-hand side of the diagram, indicating the assessment result of fine sediments corresponding to factor 1, PLI (overall contamination of the site), and contamination. Therefore, it was determined that grain size has a large influence on these four metals. Furthermore, coarse sediments (very coarse sand, coarse sand, and medium sand) have a small effect on contamination. However, as the concentrations of Cd have a relatively weak relationship with the main factor, fine sand, it is determined that Cd has a strong influence on metal contamination in the study area (Fig. 7).

**Table 5:** Component matrix (factors 1 and 2) and eigenvalue loading of surface sediments in the study area (principal component analysis)

Units		Factor 1	Factor 2	Units		Factor 1	Factor 2	Units		Factor 1	Factor 2	Units		Factor 1	Factor 2
Grain size	Very coarse sand	− 0.465	− 0.621	Metals	Al	0.291	− 0.575	EF	Zn	0.752	− 0.172	Igeo	Zn	0.772	− 0.437
	Coarse sand	− 0.631	− 0.002		Li	0.859	− 0.083		Cr	0.266	0.929		Cr	0.541	0.740
	Medium sand	− 0.145	0.785		Zn	0.777	− 0.392		Pb	0.484	0.444		Pb	0.832	− 0.025
	Fine sand	0.345	0.224		Cr	0.526	0.690		Cu	0.855	0.021		Cu	0.923	− 0.186
	Very fine sand	0.726	− 0.110		Pb	0.844	− 0.072		Ni	0.757	0.365		Ni	0.828	0.201
	Silt	0.736	− 0.275		Cu	0.879	− 0.169		Cd	0.184	0.434		Cd	0.497	0.149
	Clay	0.657	− 0.257		Ni	0.864	0.112	PLI		0.963	0.072	Eigenvalue loading		46.08%	15.08%
	Mz	0.805	− 0.023		Cd	0.395	0.022								
	So	0.785	− 0.334	-											
	Sk	0.420	− 0.296

The factors that have large correlation coefficients generally exhibit close relationships with geochemical or environmental characteristics. As sediment grains become finer, the surface area becomes larger. Consequently, more matter can be adsorbed, and the increase in ion exchange facilitates the adsorption of metals and organic matter (Horowitz 1991). Therefore, we suggest that the study area is predominantly affected by fine sediments. Moreover, because of flow rate and flow velocity effects, metal concentrations and contamination are relatively high at sites adjacent to the weirs, which are composed of fine sediments. And the highest contamination levels were found at NS19 (adjacent to the Haman weir). Therefore, structures such as artificially constructed weirs are thought to be the major factor that affect grain size distribution and control the contamination of metals in the sediments of this study area.

## Conclusions

In order to understand the contamination environment of surface sediments in the entire Nakdong River system, surface sediments were collected from 21 sites in the mainstream and tributaries of Nakdong River, South Korea. Through a grain size analysis of the collected surface sediments, the sediment composition, mean grain size, sorting, and skewness were obtained. Moreover, the contamination was assessed by analyzing the metal concentration in the sediments, comparing it with the SQGs of different countries, and calculating the EF, Igeo, and PLI values.

In the study area, sand was the dominant grain size; yet, sites adjacent to weirs were composed of relatively fine sediments. This indicated that fine sediments are deposited due to the reduced flow rate and flow velocity around weirs. A comparison of the metal concentrations with the SQGs of USA, Canada, and Korea revealed that contamination exceeded the standard concentrations at sites adjacent to weirs. EF and Igeo values also showed high levels of contamination at sites adjacent to weirs, with Cd contamination the highest. Furthermore, PLI values revealed that the highest contamination of all weir-adjacent sites occurred at site NS19, located at Haman weir.

In conclusion, anthropogenic contamination in surface sediments of the study area is extremely low, and findings revealed that metal contamination levels are influenced by the effect of fine sediments. Moreover, metal concentrations tend to accumulate substantially at sites adjacent to weirs, where fine sediments are actively deposited because of reduced flow rate and flow velocity. Among the metals, Cd concentrations exhibited a weak relationship with fine sediment content and demonstrated the greatest effect on metal contamination in the study area. Furthermore, the highest contamination by metals occurred at site NS19, which is located adjacent to the Haman Weir. Thus, the overall accumulation of fine sediments increased due to the influence of weirs, thereby increasing pollution by metal contamination in sediments of the study area.
